# P-266. Declining Prevalence of AIDS-Defining Illness Among Newly Diagnosed People Living with HIV in Northern Thailand: A 10-Year Review

**DOI:** 10.1093/ofid/ofaf695.487

**Published:** 2026-01-11

**Authors:** Ditsaruj Srijiranon, Nuttanun Wongsarikan, Romanee Chaiwarith

**Affiliations:** Chiang Mai University, Muang, Chiang Mai, Thailand; Chiang Mai University, Muang, Chiang Mai, Thailand; Faculty of Medicine, Chiang Mai University, Chiang Mai, Chiang Mai, Thailand

## Abstract

**Background:**

HIV infection and AIDS remain a serious global health problem. Previous studies in Thailand have shown that about half of newly diagnosed people living with HIV (PLWH) presented with AIDS-defining illnesses (ADIs). Data on the prevalence of ADIs, opportunistic infections (OIs) and hospitalization among PLWH in northern Thailand was sparse.

**Methods:**

A retrospective cohort study was conducted among 777 newly diagnosed PLWH between January 1, 2012 and December 31, 2022 in a tertiary care hospital in northern Thailand. The primary objective was to determine the prevalence of ADIs in newly diagnosed PLWH. The secondary objectives were to describe the clinical presentation and to determine the hospitalization rate and overall mortality in newly diagnosed PLWH.

**Results:**

The prevalence of ADIs in newly diagnosed PLWH was 29.0% (225/777), with a decreasing trend over the years (p=0.021). Of the 552 patients without ADIs, 57.2% (316/552) were asymptomatic. The common reasons for an HIV test in asymptomatic patients were unprotected sex (43.2%), an HIV-positive partner (17.0%) and pre-operative evaluation (11.0%). The common ADIs included *Pneumocystis* pneumonia (42.7%), talaromycosis (27.1%) and pulmonary tuberculosis (14.2%). The median (IQR) CD4 count at diagnosis was 34 (16-83) and 293 (183-437) cells/cu.mm in patients with and without ADIs, respectively. The hospitalization rate among newly diagnosed PLWH was 25.5% (72.0% and 6.5% in patients with and without ADIs, respectively). Only one patient died during hospitalization.

**Conclusion:**

Even in the era of cART treatment, a significant proportion of PLWH in northern Thailand are diagnosed late, emphasizing the need for enhanced screening and intervention strategies.

**Disclosures:**

All Authors: No reported disclosures

